# Advancing brain health and dementia research in Canada: insights from focus groups with the Dementia Research and Innovation Funders Alliance

**DOI:** 10.1093/geront/gnag124

**Published:** 2026-06-08

**Authors:** Juanita-Dawne R Bacsu, Alixe Ménard, Cheltey Berlinguette, Wendy Hulko, Megan E O’Connell, Sarah Fraser, Jim Mann, Sheila Blackstock, Myrna Norman, Melba Sheila D’Souza, Marc Viger

**Affiliations:** School of Nursing, Population Health and Aging Rural Research (PHARR) Centre, Thompson Rivers University, Kamloops, British Columbia, Canada; Interdisciplinary School of Health Sciences, University of Ottawa, Ottawa, Ontario, Canada; School of Nursing, Population Health and Aging Rural Research (PHARR) Centre, Thompson Rivers University, Kamloops, British Columbia, Canada; School of Social Work and Human Service, Population Health and Aging Rural Research (PHARR) Centre, Thompson Rivers University, Kamloops, British Columbia, Canada; Department of Psychology & Health Studies, University of Saskatchewan, Saskatoon, Saskatchewan, Canada; Interdisciplinary School of Health Sciences, Faculty of Health Sciences, University of Ottawa, Ottawa, Ontario, Canada; National dementia advocate and person living with dementia, Surrey, British Columbia, Canada; School of Nursing, Thompson Rivers University, Kamloops, British Columbia, Canada; National dementia advocate and person living with dementia, Maple Ridge, British Columbia, Canada; School of Nursing, Thompson Rivers University, Kamloops, British Columbia, Canada; Dr. Marc Viger Medical Professional Corporation, Population Health and Aging Rural Research (PHARR) Centre, Kamloops, British Columbia, Canada

**Keywords:** Dementia, Brain health, Implementation science, Life course, Collaboration

## Abstract

**Background and Objectives:**

Strategic planning and prioritization are critical to fueling scientific acceleration to advance dementia prevention, treatment, and quality of life. Although funding agencies and community leaders have important roles in supporting research, their insights remain largely overlooked. Recently, the Dementia Research and Innovation Funders Alliance was created by the Canadian Institutes of Health Research to foster collaboration, innovation, and identify impactful ways to mobilize research findings on brain health and dementia. Through focus groups with Alliance members, this study aimed to gather expert insight to accelerate brain health and dementia research in Canada.

**Research Design and Methods:**

Three focus groups were conducted with 20 Alliance members from June 24 to 26, 2025. The focus groups were led by an experienced moderator who asked four open-ended questions exploring priorities to maximize research impact, including translation into practice and policy. Thematic analysis was used to analyze the data.

**Results:**

Four themes were identified: (i) implementation gap: translating research into action; (ii) life-course approach: enhancing brain health promotion across all ages; (iii) global collaboration: leveraging international connections to drive research and innovation forward; and (iv) equity-denied groups: advancing inclusion and community-based research.

**Discussion and Implications:**

While extensive studies have been conducted on brain health and dementia research, there is a pressing need to translate research findings into practice and policy. It is critical that future research prioritizes implementation science across the life course, international collaboration, and partnerships with equity-denied groups to advance brain health and dementia research and innovation in Canada.

## Background and objectives

Dementia is a growing issue in Canada, with almost 10 people being diagnosed each hour (Public Health Agency of Canada [PHAC], 2024). In 2023, nearly 487,000 Canadians were living with a diagnosis of dementia (PHAC, 2025). By 2030, it is estimated that almost one million Canadians will be living with this syndrome (Alzheimer Society of Canada [ASC], 2025). The financial cost of dementia is substantial, with economic challenges at the individual, community, and broader societal levels. In 2020, the economic impact of dementia in Canada was estimated at $40.1 billion, with an average of $67,200 per person in direct and indirect costs (Canadian Centre for Economic Analysis, 2023).

In 2019, the Government of Canada launched, *A Dementia Strategy for Canada: Together We Aspire*, to address the growing impact of dementia (Government of Canada, 2019). The Strategy provided a vision to help guide future actions to advance dementia support at the individual, community, and policy level. The Strategy acknowledged research and innovation as a foundational pillar and identified three national objectives: (1) to prevent dementia, (2) improve therapies and find a cure, and (3) enhance the quality of life of people with dementia and their care partners (Government of Canada, 2019). With the financial support of the Canadian Institutes of Health Research (CIHR) and other organizations, researchers have been working to address these national objectives. Despite the development of the National Strategy, there is limited knowledge on the current landscape of brain health and dementia research in Canada.

Recently, the Dementia Research and Innovation Funders Alliance was created with the support of CIHR’s Institute of Aging to advance collaborative partnerships, support innovation, and mobilize research findings on brain health and dementia (CIHR, 2023). The Alliance consists of approximately 30 representatives of the brain health and dementia research funding community across Canada (CIHR, 2025). Its non-term-limited membership extends beyond funders to include research organizations, government agencies, non-profit and advocacy groups, and innovation-focused institutions. Through its Steering Committee and thematic working groups, the Alliance was created to assess the dementia research ecosystem, identify research gaps since the enactment of the National Strategy in 2019, and inform strategic funding investments to mobilize brain health and dementia research in Canada in response to these gaps (CIHR, 2023). Despite significant investments, the research landscape has historically been fragmented, siloed, and unevenly coordinated across funders, disciplines, and regions (CIHR, 2023, 2025). Research has often been conducted within disciplinary, institutional, or regional silos, reducing opportunities for cross-sector awareness and synthesis of the broader landscape. Accordingly, the Alliance aims to increase the collective impact of funding by creating opportunities for systematic sharing and integration of existing datasets, allowing for large-scale discovery, and improved research translation into practice and policy for better real-world impact and cross-sector collaboration (CIHR, 2023).

To guide this work and ensure that future research aligned with the Alliance’s mission in advancing brain health and dementia research, a team of researchers obtained competitive funding from CIHR to conduct a national project. This project aimed to assess the current dementia research ecosystem, identify knowledge gaps, and inform future funding directions. Accordingly, this study was part of a larger project that used various data collection methods (i.e., environmental scan, scoping review, focus groups) to analyze the brain health and dementia research landscape in Canada from 2020 to 2025. For instance, our scoping review of 275 studies revealed that there were significant gaps in the knowledge related to differences in research approaches, lack of coordination across research initiatives, and many small-scale projects with inconsistent measures (Bacsu et al., 2026).

To gain more in-depth insight, we conducted focus groups with members of the Alliance to gather their perspectives on advancing the field, particularly in shaping strategic investment and funding priorities. Despite funding agencies and community leaders’ roles in supporting brain health and dementia research, their expertise and insight remain largely unexplored. However, strategic planning and implementation are crucial to driving scientific advancement to support dementia prevention, treatment, and quality of life. Through conducting focus groups with Alliance members, this study aimed to gather expert insight to accelerate brain health and dementia research in Canada.

## Research design and methods

### Study design

This study employed a qualitative descriptive design with semi-structured focus groups to gather expert insights to advance brain health and dementia research in Canada. This qualitative design was selected because it focused not on increasing conceptual understanding, but on contributing to practice change through interpretive inquiry (Doyle et al., 2020). Virtual focus groups were selected as the data collection method because they provided the opportunity for Alliance members from across the country to come together to share their perspectives and generate insights through collective discussion. The focus groups were conducted to identify research gaps, funding priorities, and areas for innovation to advance brain health and dementia research in Canada.

### Ethics

This study was reviewed and approved by the Research Ethics Board (REB file #104391) at Thompson Rivers University. The Letter of Free and Informed Consent Information and focus group questions were emailed to participants in advance to provide adequate time and opportunity for review. The letter of information included ethics-related information that is typically included in consent forms (e.g., study purpose, voluntary participation, right to withdraw, potential benefits/risks of the study, anonymity and confidentiality measures, data storage/retention, and contact information of the principal investigator).

Prior to each of the focus groups, the principal investigator (PI and first author, J-D.R.B.), read through the Letter of Free and Informed Consent Information to the Alliance members, confirmed that the participants understood the information, and provided an opportunity to ask questions. Data confidentiality was emphasized, although participants were advised that anonymity could not be fully guaranteed due to the small, identifiable nature of the interest holder group-based format. This approach followed the Tri-Council Policy Statement: Ethical Conduct for Research Involving Humans and is commonly used for virtual data collection methods such as online focus groups and surveys (Sim & Waterfield, 2019). Each of the participant’s names and any potentially identifying information were removed from the transcripts and the dissemination materials.

### Focus group facilitator

The focus group facilitator (e.g., the principal investigator and first author, J-D.R.B.), is a PhD-trained researcher with expertise in brain health, dementia, and qualitative research. She is the founding director of an interdisciplinary research centre that specializes in dementia and rural aging research. She also co-leads a national program that works to engage people with lived experience of dementia (e.g., people living with dementia and care partners) to be meaningfully involved in research. She has conducted qualitative research (e.g., focus groups, world cafés, participant observation, interviews, etc.) for almost 25 years, with extensive experience in community-based health research. The facilitator had no prior relationship with the participants in the study. She shared her research background and the reasons for conducting the study with the participants. Her expertise in qualitative research helped to ensure an inclusive and informative dialogue process throughout the focus group sessions.

### Study participants

The study participants consisted of members of the Dementia Research and Innovation Funders Alliance. The Alliance was created in 2023 as part of the CIHR Brain Health and Cognitive Impairment in Aging (BHCIA) Research Initiative to promote engagement between research funders in Canada to fuel impact in dementia research through collaboration (CIHR, 2025). This group consists of research funders and other partners such as organizational representatives and community leaders who focus on advancing brain health and dementia research in Canada. More specifically, the following 30 organizations are represented in the Alliance: Amyotrophic Lateral Sclerosis (ALS) Society of Canada, Alzheimer Society of Canada, Alzheimer Society of British Columbia, Alzheimer Society of Montreal, Alzheimer Society of Ontario, Azrieli Foundation, Brain Canada Foundation, Canadian Brain Research Strategy, Canadian Centre for Caregiving Excellence, Canadian Coalition for Seniors Mental Health, Caregiver Crosswalk Inc., Centre for Aging + Brain Health Innovation, Canadians for Leading Edge Alzheimer Research, CIHR-Institute of Aging, Fonds de Recherche du Québec—Santé, Healthcare Excellence Canada, Heart and Stroke Foundation, HelpAge Canada, Huntington Society of Canada, Krembil Foundation, National Research Council Canada, Natural Science and Engineering Research Council, Ontario Brain Institute, Ontario Ministry of Health, Parkinson Canada, Public Health Agency of Canada, Reena, Research Manitoba, Weston Family Foundation, and the Women’s Brain Health Initiative (CIHR, 2025).

Three focus groups were conducted that each involved 6-8 participants for a total of 20 participants (16 women, 4 men, representative of the Alliance’s gender distribution). This number of participants has been deemed optimal for idea generation while ensuring each participant’s voice is heard (Baxter et al., 2015). The focus groups were conducted in English. Participants were recruited through an email to the Alliance members, and there were no exclusions beyond the requirement of membership with the Alliance. Due to the small number of members in the Alliance group (e.g., 30 people), the collection of socio-demographic data (e.g., ethnicity, race, age) was not undertaken to help ensure participant anonymity of the study’s participants (e.g., 20 people). No participants dropped out of the study.

### Data collection

Semi-structured focus groups were conducted virtually using the online communication platform Zoom (2025) between June 24 and 26, 2025. Each session lasted approximately one hour in length and was facilitated by the study’s principal investigator (PI, J-D.R.B.). The focus groups were guided by four open-ended questions that were developed with suggestions and insight from the research team. These four questions included: (i) what do you think are the most significant gaps in brain health and dementia research in Canada?; (ii) what do you think are the priority areas for funding brain health and dementia research in Canada?; (iii) where do you see the field of brain health and dementia research in Canada advancing in the near future?; and (iv) how are funding agencies addressing the unique needs of equity-denied communities in dementia research, particularly in terms of supporting health equity? These questions enabled group discussion where participants were able to share their unique insights and collectively brainstorm ideas to support brain health and dementia research in Canada.

### Data analysis

Data from the focus groups were recorded and transcribed using Zoom (Zoom, 2025). The transcripts were reviewed for any inaccuracies and manually corrected by the PI. The transcripts were analyzed using thematic analysis and managed in ATlas.ti software (ATlas.ti, 2023). Drawing on Braun and Clark’s (2021) framework, inductive thematic analysis was conducted to analyze the data. Inductive thematic analysis was selected since the initial codes were created based on the data rather than a theory or framework (Braun & Clark, 2021).

The thematic analysis was conducted by two members of the research team who were well trained and experienced in conducting thematic analysis through prior qualitative research using thematic analysis. For example, one of the coders (J-D.R.B.) teaches research methods courses that include thematic analysis and has delivered academic training workshops on conducting thematic analysis based on Braun and Clarke’s (2006) framework. The second coder has collaborated with the first author on multiple studies employing thematic analysis.

**Figure 1:**
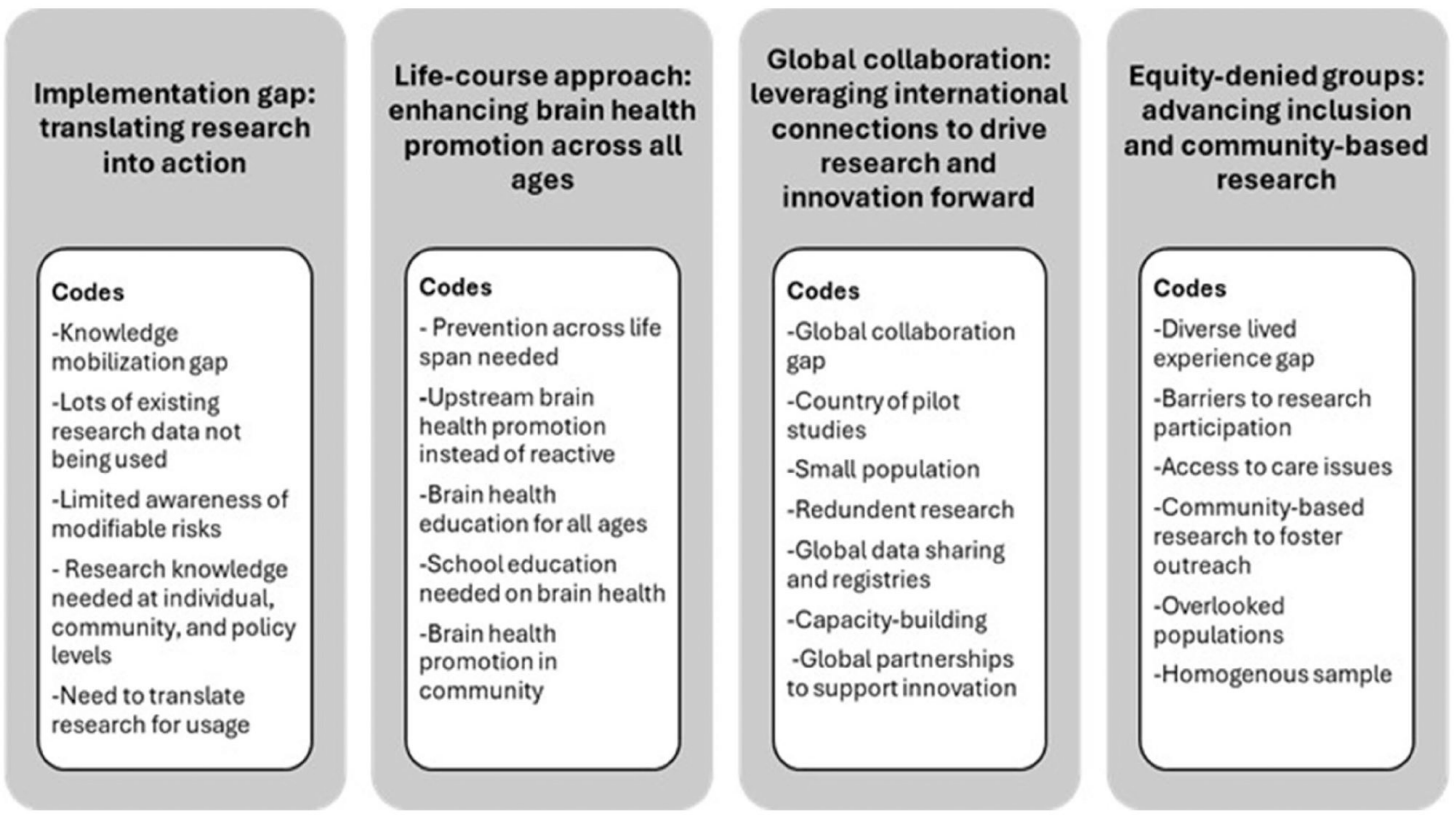
Thematic map.

The thematic analysis was conducted by the researchers iteratively reading and re-reading the transcripts to become fully immersed and familiar with the data. Initial coding was then performed where blocks of meaningful text were highlighted, and codes were created. The codes provided labels that were assigned to units of meaningful text within the transcripts such as key concepts, ideas, and challenges. Any disagreements or discrepancies in coding were resolved through open discussion and communication between the coders. For example, when one coder categorized a response as an “barriers to research participation” and another as “access to care issues,” the researchers collaboratively discussed the context and agreed on the most appropriate code. Once the coding was completed, the codes were grouped based on their similarities, patterns, and differences to create overarching themes. Our theme development process was guided by Braun and Clark’s (2006) theme generation questions. For example, we discussed the coherence of our themes, whether the data supported our themes, and whether we were missing any themes (Braun & Clarke, 2006). Additionally, we created a thematic map to document our process of grouping our codes into the development of the four themes (refer to Figure 1).

### Trustworthiness: credibility, confirmability, and dependability

To ensure trustworthiness, we employed Lincoln and Guba’s (1985) measures of credibility, confirmability, and dependability. Credibility was supported by peer debriefing with members of the research team during the thematic analysis process to help avoid issues of researcher bias and misinterpretation. Additionally, member checking was conducted by sharing the main findings from the focus groups with members of the Alliance at the Annual Dementia Research and Innovation Funders Alliance meeting held in November 2025. Confirmability was enhanced by using a range of reflexive practices (e.g., ongoing discussions within the research team, critical examination of assumptions, and data analysis by a diverse group of interdisciplinary team members representing the fields of psychology, social work, nursing, family medicine, and population health) to minimize potential bias in data interpretation. To reduce the potential for academic bias and provide a more holistic perspective based on lived experience, our research team included two people currently living with dementia who aided with the development of the focus group questions, thematic analysis, and manuscript review. It is important to note that the team members living with dementia are national advocates who are inspired to improve dementia education and awareness to improve the quality of life of people with dementia. Dependability was supported by using a strong audit trail where detailed notes were kept to document the focus group process, code development, thematic analysis framework, and decisions made during the thematic analysis process.

## Results

Based on our thematic analysis, four themes were identified: (i) implementation gap: translating research findings into action; (ii) life-course approach: enhancing brain health promotion across all ages; (iii) global collaboration: leveraging international connections to drive research and innovation forward; and (iv) equity-denied groups: advancing inclusion and community-based research.

### Implementation gap: translating research findings to action

A major theme identified throughout the focus groups was the challenge of implementing evidence-based research knowledge into action to support brain health and dementia. Participants described that while research has been conducted, there is a paucity of findings being translated into action to support brain health and dementia. More specifically, they noted that there is a lack of research focused on implementation or translating knowledge into usable practice at the individual and community levels. This disconnect between research findings and usable actions is highlighted in the following quotes:

*…This implementation gap, we have a lot of data. There is a lot of information. And making sure that information is getting utilized in the most effective way… There is a major innovation to implementation gap.* (FG-2, Participant 12)

*I think there’s that gap… between what we know and what the evidence says and what actually gets implemented and practiced.* (FG-1, Participant 3)

In discussing the implementation gap, there was a strong focus on addressing modifiable risk factors and prevention at the population level. For example, participants noted a disconnect in public awareness and education about modifiable risk factors and brain health promotion. This issue led to discussion on the need for implementation expertise among researchers to support both uptake and impact. The intersection of the implementation issue and modifiable risk factors is illustrated in the following quotes:

*There’s research being done… on modifiable risk factors, but I think there is not necessarily expertise on how… to implement them in a way that is engaging for the population we’re trying to target.* (FG -2, Participant 13)

*I think a lot of people don’t know of the modifiable risk factors… So I would say like really lack of awareness about brain health overall.* (FG-2, Participant 6)

*I think we have a good body of knowledge around prevention but I think the challenge is then how do we see that implemented into policy.* (FG-1, Participant 5)

Participants emphasized the need for targeted strategies to address implementation gaps, highlighting the importance of building implementation science expertise, increasing investment in knowledge translation, and developing approaches that engage communities and improve public awareness of modifiable risk factors.

### Life-course approach: enhancing brain health promotion across all ages

Another theme identified was the need for brain health promotion across the life course. Participants discussed that brain health education should be provided to everyone, including children, rather than waiting until someone has dementia. More specifically, participants emphasized the need to educate people about the modifiable risk factors to support brain health across the life course. In particular, participants discussed the need to educate parents and children about ways to reduce risk of concussions. This theme of brain health promotion is captured in the following quotes:

*Modifiable risk factors need to become Canada’s food guide from the time that kids enter school. Like brain health education has to be education to parents about also the risk of concussions, multiple concussions, sports education.* (FG-2, Participant 6)

*Need to focus on bringing the entire lifespan to the table rather than focusing solely on when you begin the disease and thinking about those factors, like biological factors or social factors, environmental factors, everything that would contribute to the development of dementia across the life course and how we can think about, um, modifying those…* (FG-3, Participant 20)

*In terms of gaps, I think there is a lot of research that has been done, but we are not linking it to the lifespan in terms of research on brain health… to see where we can act in terms of prevention, in terms of condition of life, and environmental factors in the life span…* (FG-3, Participant 19)

*Getting people to understand that cognitive impairment and dementia are not necessarily just those who are over the age of 65… So instead of a curative sort of reactive approach, we need to be responsive and focus on things earlier. Definitely a more upstream approach.* (FG-3, Participant 14)

A participant described the need for brain health education to be integrated into annual check-ups similar to physical exams and annual eye exams. For example, they suggested that:

*Brain health education really should become part of everyone’s annual physical checkup. We go in and we get our ears checked and our eyes checked, but nobody’s checking our brain health. You only check your brain health if it’s the memory is a concern. So, my hope is that brain health becomes part of the annual physical checkup for everyone*. (FG-2, Participant 6)

### Global collaboration: leveraging international connections to drive research and innovation forward

The need for global collaboration was central to discussions on advancing Canadian research and innovation on brain health and dementia. Focus group participants described the need to collaborate beyond national borders and join forces to build innovative capacity, enhance machine learning and artificial intelligence technology, and scale-up beyond pilot project status. Participants spoke about there being an underinvestment in platform technologies and data infrastructure and that there is a need to look beyond our borders for collaboration. This theme of global collaboration to advance innovation in brain health and dementia research is highlighted in the following quotes:

*As a nation, we are too small and we hear this thing about pilot projects that start and die in Canada and perhaps we don’t have to rebuild the wheel…* (FG-3, Participant 14)

*It would be great to see international collaborations. I feel that if researchers don’t collaborate the way they should, especially internationally, and it would be great to see sort of a breakthrough in that area, which could lead to much more development… I think on that vein, like open science and data sharing…* (FG-1, Participant 1)

*I think Canada is too small to actually do anything on our own. Our population is too small. We have to … figure out how we can ensure that the data that we’re all collecting can be… shared and explore ideas like federated learning, for example, is a way to ensure that data never has to leave Canadian borders but can be co-mingled with other datasets and to be able to then bring new machine learning tools and algorithms in a way that doesn’t compromise PHI [protected health information] and at the same time also doesn’t compromise any IP [internet protocol address]. But I think it requires us to look up beyond our borders instead of trying to figure out what Canada can do on its own because, again, we’re just too small of a population.* (FG-3, Participant 15)

*I would totally echo that point about how do we leverage our strengths as Canadians, as collaborators, as people who can bring people together and create opportunities for international researchers.* (FG-3, Participant 17)

Participants also discussed the need for opportunities to share experiences and data worldwide in an effort to join forces in brain health research. A clear need was identified to create data registries that support global collaboration and lead to best practices that could be adapted to the Canadian context. This need for international collaboration to share knowledge and data registries is highlighted in the following quotes:

*We need to have some kind of inventory or registry to know who’s working on what and what are strengths in Canada, and then say what is [the] UK working on, what is Japan working on, and then you can join forces depending on your strength or your interests and everything. I think it’s really valuable to work collaboratively, worldwide, and sharing experience and data, and I think that will be really helpful.* (FG-3, Participant 19)

*There’s lots of great innovation that’s happening internationally on best practices and best policies that we can build on and tailor to a Canadian context.* (FG-3, Participant 14)

### Equity-denied groups: advancing inclusion and community-based research

Another theme identified was the need to prioritize equity-denied groups to advance brain health and dementia research in Canada. For example, participants recognized that there was still a strong predominance of studies focusing on Caucasian, well-educated, urban-dwelling people in dementia research. Participants underscored the importance of having representation from more diverse population groups in brain health and dementia research. Specifically, participants noted that homogeneity in research participants was problematic in that it limited the generalizability and representativeness of research findings, especially among populations often overlooked in dementia research and equity denied groups (e.g., barriers based on ethnicity, disability and intellectual disabilities, age, early onset dementia, traumatic brain injury, geography, economic status, Indigeneity, gender identity and gender expression, nationality, race, sexual orientation, people living with down syndrome, etc.). This gap in participant diversity and the need to include more heterogeneity in dementia research is highlighted in the following quotes:

*We still have a very white, homogenous, well-educated affluent population…we see that with the disease modifying therapies that are coming out.* (FG-1, Participant 3)

*Not having full representation of the population is definitely an area of weakness… even just understanding and knowing that there’s differences between males and females who have dementia and how much more aggressive a lot of these symptoms are in women. And yet that is not even being portrayed accurately…* (FG-2 – Participant 13)

*Exclusion of equity-deserving groups in dementia research, [where] available treatments and diagnostics are specifically tailored to…Western educated, industrialized, rich and democratized populations.* (FG-3, Participant 16)

Participants described the need for research to examine the barriers to research participation among equity denied groups. In particular, participants addressed the need to understand the challenges of dementia diagnosis, dementia care, and the meaning of quality of life. The need to understand barriers to diverse research participation is highlighted in the statements below:

*I think we should talk about health equity in research, [and] what’s happening so we can understand the barriers related to diagnosis of disease, care, research participation, especially in areas that are more rural, maybe Indigenous, Francophone…* (FG-2, Participant 8)

*…start reducing some of those barriers to participation and start expanding the portfolio who’s getting engaged in dementia research beyond the white, wealthy, well -educated, urban people who might be easy to reach. I think there’s some opportunities there.* (FG- 2, Participant 12)

*I’m just gonna echo the things about bringing the research to the community… And it does really help that issue around how do you ensure that vulnerable populations are included is that often in clinical settings they may be very intimidat[ing]… So bringing it to the community and then being adaptive and accommodating to ensure people are included in research, and also capture that piece around what does quality of life mean to you.* (FG-3, Participant 17)

*Improving access to care for people in rural areas and those that have less physical access to care…* (FG-3, Participant 16)

Community-based research approaches were identified as a key strategy to engage more diverse groups in brain health and dementia research. In particular, participants shared that research partnerships and outreach should be conducted at the community-level. For example, they noted the importance of using a “boots on the ground” approach by conducting collaborative research in partnership with the communities rather than using a top-down approach. This community-based approach to foster more diverse engagement in research is illustrated in the following quotes:

*Shift towards engaging in team-based research that is of equal nature…engagement of different ethnic groups, socio-economic, and cultural groups in the community… that’s an area that we have to work on…* (FG-2, Participant 7)

*Research doesn’t make it to [people with dementia]. I call it boots on the ground. I don’t feel like researchers have boots on the ground to the level that it should be… We can’t just say, let’s get on Zoom. Get off zoom. Invest sometime to go into the community… Researchers have to put their faces out there… A lot of this is, who are you? Do we trust you? Do we feel like you are really trying to help us?* (FG-3, Participant 18)

*I think any kind of partnership models where we can take our lead directly from the communities that we’re trying to serve rather than going top down is also a really helpful way to support equity enhancing research.* (FG-2, Participant 12)

Participants noted the importance of hands-on and team-based approaches that include direct community engagement in the research, including partnerships with local organizations, rather than solely working at an institutional level using top-down approaches.

## Discussion and implications

Dementia is a growing concern for many Canadians (Fasoro et al., 2025). Using focus groups with members of the Dementia Research and Innovation Funders Alliance, the purpose of this study was to gather expert insights to advance brain health and dementia research in Canada. Four main themes were identified and provided insights into a need to close the implementation gap between evidence and practice; promote brain health across the life course through early and ongoing education; strengthen global collaboration and data infrastructure; and advance equity through inclusive, community-based research that meaningfully engages equity-denied populations.

Our study’s findings identified a critical implementation gap in the translation of research findings into action, especially in terms of prevention and modifiable risk factors to promote brain health. Our participants noted that despite an extensive body of research on dementia prevention, gaps remain in its implementation into policy and practice. This finding is supported by existing literature that has documented knowledge gaps in supporting brain health promotion at the individual, community, (James et al., 2025) and policy levels (Lancet Neurology, 2026). For example, existing research has shown that brain health promotion and education on modifiable risk factors have rarely been communicated to the public (Daly, 2025; Meng et al., 2024; Ownby & Caballero, 2025; PHAC, 2019); healthcare providers historically lacked the confidence, training, and resources to diagnose and support quality of life of people living with dementia (Bacsu et al., 2020; Canadian Institute for Health Information, 2016; Travers Altizer et al., 2025); and persistent dementia-related stigma, fear, and misinformation undermines access to community supports (Bacsu et al., 2022, 2024; Siette et al., 2023). Moreover, there is a critical need to make brain health a priority for policy through continued investment and national leadership (Lancet Neurology, 2026). Addressing these gaps will require strengthening knowledge translation mechanisms, such as embedding evidence-based guidelines into clinical practice, supporting continuous brain health promotion and dementia education, and enhancing partnerships to ensure that existing knowledge is accessible, relevant, and actionable at the individual, community, and policy levels.

Findings from our study emphasized that research needs to target brain health education and awareness across the life course rather than waiting until after a dementia diagnosis. This finding aligns with existing research that shows that neurodegeneration demands a life-course approach given the multiple decades between exposure and outcome (James et al., 2025). Moreover, this finding is supported by an emerging body of literature that is calling for the inclusion of younger and middle-aged populations in brain health promotion and dementia prevention efforts (Farina et al., 2024; Laver et al., 2025; Ownby & Caballero, 2025). However, there is a lack of actionable guidance to facilitate brain health promotion at the population level (Ownby & Caballero, 2025). Consequently, it is essential that future studies prioritize implementation strategies and knowledge mobilization activities to ensure the uptake of preventative measures and modifiable risk factors across the life span.

Our study’s findings highlighted the importance of international collaboration and global partnerships for building capacity to fuel dementia research and innovation in Canada. This finding is reflected by previous calls for national and international collaboration between researchers to exchange ideas and prevent the duplication in dementia research (Morello-Garcia et al., 2025). Of note, the Government of Canada recently invested $1.7 billion in the *Canada Impact+ Research Talent Initiative* in an effort to attract international researchers to Canada for the purpose of bolstering international collaboration and partnering in global innovations (Government of Canada, 2025). This effort has the potential to strengthen the brain health research ecosystem at the height of current global health challenges related to dementia. Global nonprofit coalitions are also important interest holders in bolstering international partnerships, such as the Alzheimer’s Disease Data Initiative (e.g., AD Data Initiative) that recently developed a secure, cloud‐enabled platform to support global collaboration with access to data sharing, analysis of datasets, and seamless integration with other major data platforms.

In the global context, dementia research funding has historically been distributed across multiple organizations, countries, and disciplines without strong coordination mechanisms (Morello‐Garcia et al., 2025). This led to the duplication of research efforts, gaps in key priority areas, and inefficient use of limited funding (Zhu et al., 2026). In response, initiatives like the International Alzheimer’s and Related Dementias Research Portfolio (IADRP, n.d.) were created to map funding, identify overlaps, highlight gaps, and enable funders to better coordinate investments. This global example directly reflects the Alliance’s mission to improve transparency and compile data from all Canadian-funded dementia research (CIHR, 2023). Large-scale international partnerships, such as the Joint Programme—Neurodegenerative Disease Research (JPND, 2020) and the World Wide Alzheimer’s Disease Neuroimaging Initiative (WW-ADNI) (Weber et al., 2021), have also been developed to foster knowledge sharing and support coordinated research agendas across multiple countries. Collaborative efforts like the WW-ADNI have shown promise by openly sharing data and standardizing analyses for foundational Alzheimer’s disease treatment trials, and amyloid and tau phenotyping across the globe (Weber et al., 2021).

The findings from our focus groups identified the need for more inclusion and diversity in brain health and dementia research. More specifically, the participants in our study emphasized that dementia research is not representative of Canada’s diverse population with limited demographic, cultural, linguistic, and geographic diversity. This finding is consistent with nascent research that dementia studies lack representation from diverse groups that typically face significant inequity and greater risk of dementia (Bacsu et al., 2025; Maestre et al., 2024). Consequently, there is a need to de-homogenize study populations and design dementia studies with diverse, representative populations, and contextualized interventions that can be adapted with different groups at the population level (Woltering et al., 2019). Scaling interventions successfully requires consideration of sociodemographic diversity, local infrastructure, and cultural factors, ensuring that findings are generalizable and that interventions are effective for populations that have historically been excluded from research (Koorts et al., 2025). Ahmed and colleagues (2025) note that dementia clinical trials are often biased from recruitment of homogenous patients with little representation of diversity which limits the generalizability of the studies findings. Accordingly, there is a crucial need for future research to understand participation barriers and inclusion strategies to enhance diversity in research, especially among equity-denied groups.

Overall, our study’s findings shed light on the urgent need to translate brain health and dementia research into actionable strategies that reach diverse populations across the lifecourse. While continued investment in research is essential, equal emphasis must be placed on enabling practitioners, public health agencies, and educators to apply existing evidence to real-world settings through scalable interventions at the individual, community, and policy levels. The themes identified in our study have broad relevance across gerontology, as they reflect systemic challenges and emerging priorities that extend beyond dementia to aging-related research. More specifically, our findings emphasized the need to strengthen how evidence is generated, translated, and applied to support healthy aging across diverse populations and contexts, with particular focus on early upstream approaches and brain health promotion across the life course. For example, integrating brain health into school curricula or routine primary care has parallels with broader efforts to embed healthy aging principles into education systems and preventive healthcare. Further, the need for international collaboration is directly applicable to other domains in gerontology such as comparative aging policy, long-term care models, and age-friendly environments, making data sharing and coordinated infrastructure of increased relevance. Advancing this agenda will require sustained investment in implementation science, global collaboration, and community-based approaches that prioritize equity and inclusivity, ensuring that knowledge benefits all segments of society.

### Limitations

Although our study was conducted in a rigorous manner, it is not without limitations. Given that our focus groups only included members from the Dementia Research and Innovation Funders Alliance, our findings may not be representative of other groups’ perspectives on priorities such as community dwelling people living with dementia, community leaders, researchers, and healthcare professionals outside of the Alliance. Accordingly, future research is needed to explore the brain health and dementia research priorities from the perspectives of community leaders, healthcare professionals, and people with lived experience, including people from equity-denied groups.

## Conclusion

This study was conducted with members of the Dementia Research and Innovation Funders Alliance to identify ways to advance brain health and dementia research in Canada. Four themes emerged ranging from the need for research with equity-denied groups to international collaboration to support research and innovation. Findings from our study shed light on priority areas to strengthen brain health and dementia research in Canada. Participants emphasized the persistent gap between research evidence and its translation into policy and practice, alongside the need for prevention-focus approaches across the life course, greater global collaboration, and more inclusive research practices. In future studies, it is essential to prioritize implementation and knowledge mobilization to ensure that evidence-based findings are enacted at the population level. Specifically, targeted knowledge mobilization, funding supports, and implementation strategies are needed to ensure uptake and usage of modifiable risk factors to enhance brain health promotion across the life course. Moreover, international partnerships and collaboration are needed to build capacity to advance dementia research and innovation. Additionally, there is a critical need to prioritize equity-denied groups and understand barriers to improving their research participation. Moving forward, it is critical that future research prioritizes implementation science across the life course, partnerships with equity-denied groups, and international collaboration to advance brain health and dementia research in Canada.

## Data Availability

(1) Supplementary materials (list of codes) are available to other researchers for replication purposes by contacting the lead author; (2) supplementary data are not available, as the small size of the Alliance membership presents a risk to the participants’ confidentiality and anonymity; and (3) the study reported in the manuscript was not pre-registered.
